# The influence of ABCG2 polymorphism on erlotinib efflux in the K562 cell line

**DOI:** 10.1002/prp2.581

**Published:** 2020-04-07

**Authors:** Anna Svedberg, Lianne Jacobs, Svante Vikingsson, Henrik Gréen

**Affiliations:** ^1^ Clinical Pharmacology Division of Drug Research Department of Medical and Health Sciences Linköping University Linköping Sweden; ^2^ Department of Forensic Genetics and Forensic Toxicology National Board of Forensic Medicine Linköping Sweden

**Keywords:** ABCG2, erlotinib, LC–MS/MS, polymorphism, transport

## Abstract

Single nucleotide polymorphisms (SNPs) in the gene for multidrug resistance protein ABCG2, an erlotinib transporter, is a possible contributor to the interindividual variation observed in erlotinib pharmacokinetics and toxicity. Therefore, the aim was to study erlotinib efflux by ABCG2 wild‐type (wt) and ABCG2 polymorphic variants in the K562 cell line. The chronic myeloid leukemia K562 cell line, neither expressing EGFR nor ABCG2, was transduced with vectors containing the *ABCG2* wt, the SNPs: 34 G > A and 421 C > A, or with empty vector (K562/ve). ABCG2‐expressing cells were enriched using magnetic sorting and the expression was verified using flow cytometry. Intracellular erlotinib concentrations were analyzed by LC–MS/MS after incubation with 1 µmol/L erlotinib for 60 minutes. All recombinant cell lines were confirmed carriers of the vector and expressed ABCG2. Differences in intracellular erlotinib concentrations were observed between K562/ve and K562 *ABCG2* wt and between K562/ve and K562 *ABCG2* 34G > A (both *P* ≤ .001, one‐way ANOVA with Tukey HSD post hoc test), indicating that the cell lines carrying *ABCG2* wt and *ABCG2* 34G > A actively transports erlotinib out of the cells. The *ABCG2* 34G > A cell line had a higher transport capacity compared with *ABCG2* wt after adjusting for ABCG2 expression (*P* = .024, *t* test). No differences were observed between K562/ve and K562 *ABCG2* 421 C > A. Genetic polymorphism in the ABCG2 gene has an influence on the transport of erlotinib which can contribute to the observed variation in erlotinib pharmacokinetics and toxicity.

**Abbreviations**
ABC: ATP‐binding cassette
ACN: acetonitrile
AmAc: ammonium acetate
BCRP: breast cancer resistance protein
EGFR: epidermal growth factor receptor
EYFP: enhanced yellow fluorescent protein
FBS: fetal bovine serum
HPLC: high‐performance liquid chromatography
LC–MS/MS: liquid chromatography‐tandem mass spectrometry
MDR: multidrug resistance
MeOH: methanol
MFI: mean fluorescence intensity
NSCLC: non–small cell lung cancer
PBS: phosphate‐buffered saline
SNP: single nucleotide polymorphism
TKI: tyrosine kinase inhibitor
wt: wild type

## 1 INTRODUCTION

The epidermal growth factor receptor (EGFR) tyrosine kinase inhibitor (TKI) erlotinib is used in the treatment of non–small cell lung cancer (NSCLC) patients harboring somatic EGFR mutations, including exon 19 deletion and exon 21 L858R substitution.^1^

Erlotinib is a targeted therapy that is administered orally once daily. Large interindividual variation is observed in erlotinib pharmacokinetics. Plasma drug concentrations have been associated with adverse drug reactions,^2^ which occurs in up to 75% of the patients, and overall survival.^3^, ^4^ Additional understanding of the underlying variation in pharmacokinetic parameters involved in erlotinib treatment would be of interest. One possible contributor could be the variability in the activity of the transporter and multidrug resistance‐associated protein ABCG2.

The membrane‐associated protein ABCG2, also known as the breast cancer resistance protein (BCRP), belongs to the ATP‐binding cassette (ABC) transporter family Horsey Aaron et al.^5^ ABCG2 is a half‐transporter that utilizes ATP hydrolysis to extrude endogenous substances as well as xenobiotics out of the cell by efflux.^6^ Naturally, ABCG2 expression is prevalent in the small intestine, colon, liver, stem cells, and barrier tissues including brain, placenta, and testis.^7^, ^8^, ^9^, ^10^ The protective function of ABCG2 is also utilized by cancerous cells. In chemotherapy treatment, the overexpression of ABCG2 in tumors is associated with multidrug resistance (MDR) which leads to increased efflux and reduced intracellular accumulation of drugs in cancer cells Horsey Aaron et al.^5^

Concentrations of erlotinib in tumors are found to vary between <5 nmol/L and 1.1 µmol/L.^11^ The 40‐fold variation in erlotinib tumor concentration might be due to large interindividual variability in transport activity. This variability in efflux might be caused by natural genetic variation in the ABCG2 gene which in turn can affect the ABCG2 efflux activity.

In general, the *ABCG2* nonsynonymous single nucleotide polymorphisms (SNPs) 34 G > A (V12M) and 421 C > A (Q141K) are vastly studied. The *ABCG2* 421C > A polymorphism is known to generate a lower protein expression compared to *ABCG2* wild type (wt),^12^ while *ABCG2* 34 G > A shows expression levels comparable to *ABCG2* wt.^13^

Earlier ABCG2 in vitro studies with erlotinib have determined that erlotinib is an ABCG2 substrate and that ABCG2 wt is more efficient in transporting erlotinib compared to control cell lines.^14^, ^15^ Additionally, erlotinib intracellular concentrations in an *ABCG2* 421 C > A carrying cell line were not different compared to the control cell line.^14^ How erlotinib is transported by ABCG2 carrying the 34 G > A remains to be studied.

Therefore, we aim at investigating how *ABCG2* wt and *ABCG2* polymorphisms influence the intracellular concentrations of erlotinib after drug exposure. Intracellular accumulation of erlotinib was assessed in the chronic myeloid leukemia cell line, K562, stably transduced with *ABCG2* wt, the *ABCG2* polymorphisms 34 G > A and 421 C > A, and empty vector. By using a non–EGFR‐expressing leukemic cell line, the effect of erlotinib on cell survival is minimized.

## 2 MATERIALS AND METHODS

### 2.1 Drugs and chemicals

Reference substances for erlotinib and erlotinib‐D6 were purchased from Toronto Research Chemicals. Acetonitrile (ACN) of LC–MS grade was purchased from Merck AB and methanol (MeOH) of HPLC grade from Thermo Fischer Scientific. Ammonium Acetate (AmAc) of analytical grade was obtained from Sigma Aldrich.

### 2.2 Cell cultivation

The parental K562 cell line (ATCC‐LCG Standards) and the transduced K562 cell lines were cultivated in RPMI 1640 medium supplemented with 10% fetal bovine serum (FBS) and 2% penicillin/streptomycin at 37°C with 5% CO_2_. The cells were maintained by replacement or addition of fresh media to a concentration of 200 000 cells/ml every 3‐4 days. Cell culture reagents were obtained from Life Technologies. All cell lines were tested negative for mycoplasma infection.

### 2.3 Transduction of the K562 cell line

The K562 cell line was considered to be a suitable modeling system to study how erlotinib is transported by ABCG2 as K562 naturally neither express ABCG2 nor EGFR (http://www.proteinatlas.org).^16^ SNPs considered for selection were nonsynonymous SNPs, not resulting in a stop codon and with a minor allele frequency (MAF)> 0.02 in any population. The two selected SNPs, 34 G > A, and 421 C > A were predicted to be tolerated by SIFT and Poly‐Phen^17^, ^18^ and are present in >10% of the total population, Table 1.

**TABLE 1:** Summary of the studied ABCG2 polymorphisms

SNP	Amino acid change	rsID	Protein location	SIFT		Poly‐Phen		MAF^a^ (total)
				Score	Prediction	Score	Prediction	
34 G > A	V12M	rs2231137	Intracellular	1.00	Tolerated	0.00	Benign	0.1101
421 C > A	Q141K	rs2231142	Intracellular	0.33	Tolerated	0.37	Benign	0.1493

Abbreviation: MAF, minor allele frequency.

^a^ Total MAF obtained from gnomAD dataset v2.1.1.

K562 was stably transduced with *ABCG2* wt, the ABCG2 polymorphisms 34 G > A and 421 C > A, and empty vector as previously described.^19^ Briefly, *ABCG2* was cut out of the pCMV6‐XL5 *ABCG2* vector and ligated to MSCV‐IRES‐enhanced yellow fluorescent protein (EYFP) retroviral vector (MIY) by utilization of Not1 restriction enzymes. 293T cells were transfected with MIY‐*ABCG2* and helper vectors VSVG and POL‐GAG following transduction of K562 using spin infection of the collected viral 293T supernatant. An empty MIY vector was transduced into K562 to serve as a control cell line.

The final recombinant K562 cell lines were a control cell line with MIY vectors without *ABCG2* (K562/ve), a cell line with *ABCG2* wt (K562 *ABCG2* wt), and cell lines with the *ABCG2* variants 34 G > A (K562 *ABCG2* 34) and 421 C > A (K562 *ABCG2* 421).

### 2.4 ABCG2 and EYFP expression

Enrichment of ABCG2‐expressing cells was performed using magnetic sorting with microbeads conjugated to monoclonal anti‐human ABCG2 (CD338) antibodies (Miltenyi Biotec). Briefly, 1 × 10^7^ cells were labeled with microbeads and separated on an LS column with a magnetic separator.

Expression of ABCG2 and EYFP was quantified using flow cytometry (Beckman Coulter) after extracellular staining ABCG2 with PerCP‐Cy™5.5 Mouse Anti‐Human CD338 antibody obtained from BD Biosciences. Before analysis, 1 × 10^6^ cells were labeled with the ABCG2 antibody and washed twice with phosphate‐buffered saline (PBS).

### 2.5 Intracellular accumulation

The cell lines were seeded at 400 000 cells/mL in a total volume of 5 mL and incubated with a final concentration of 1 µmol/L erlotinib. After 60 minutes of incubation at 37°C with 5% CO_2_ the cells were transferred to a 15 mL tube and centrifuged at 1000*g* for 5 minutes at room temperature and washed twice with 5 mL cold PBS. The cells were lysed with 200 µL of MeOH containing 6.25 ng/mL erlotinib‐d6 and 200 µL of 10 mmol/L AmAc. The samples were thoroughly vortexted for 10 seconds before centrifugation at 17 000*g* for 10 minutes at 4°C. The supernatant was transferred to a 96‐well plate and stored at −20°C before LC–MS/MS analysis. Two independent experiments were carried out in triplicates for all cell lines except for K562/ve that contained one replicate in the second experiment.

The ABCG2 transport capacity was determined by normalizing the intracellular concentration of erlotinib using the ABCG2 expression. The difference in intracellular erlotinib concentration between the average K562/ve and a replicate from an ABCG2‐expressing cell line was divided by the PerCP‐Cy5.5 response from the ABCG2‐expressing cell line adjusted for isotype expression (Δ ng/mL/Δ MFI).

### 2.6 LC–MS/MS analysis

Erlotinib and erlotinib‐d6 were analyzed using a previously described LC–MS/MS method.^20^ Briefly, erlotinib (m/z = 395.2‐279.2) and erlotinib‐d6 (m/z = 400.2‐292.1) were monitored on an Aquity Xevo triple quadrupole mass spectrometer (Waters) in a positive mode after separation on an XBridge C18 column (100 × 2.1 mm, 1.7 µmol/L, Waters) at 55°C on an Acquity UPLC System (Waters). The total run time was 7 minutes with a gradient of 5 mmol/L AmAc and ACN, 10%‐50% ACN 0‐5 minutes, followed by 90% ACN 5‐6 minutes and 10% ACN 6‐7 minutes.

Calibration and quality control samples for erlotinib were separately prepared at 25, 75, 375, 1200, 375,0 and 5000 ng/mL and, 25, 75, 400, 1000, and 3750 ng/mL in 50% MeOH, respectively. To 50 µL of each sample, 200 µL of MeOH containing 6.25 ng/mL erlotinib‐d6 and 200 µL of 10 mmol/L AmAc was added. The samples were transferred to a 96‐well plate and stored at −20°C before LC–MS/MS analysis.

### 2.7 Statistical analysis

Statistical differences in intracellular concentrations between cell lines were analyzed with one‐way ANOVA and Tukey HSD post hoc test and differences in transport capacity normalized for ABCG2 expression were analyzed with Student's independent *t* test using the IBM SPSS Statistics, version 26 (IBM).

## 3 RESULTS

### 3.1 ABCG2 expression

**FIGURE 1:**
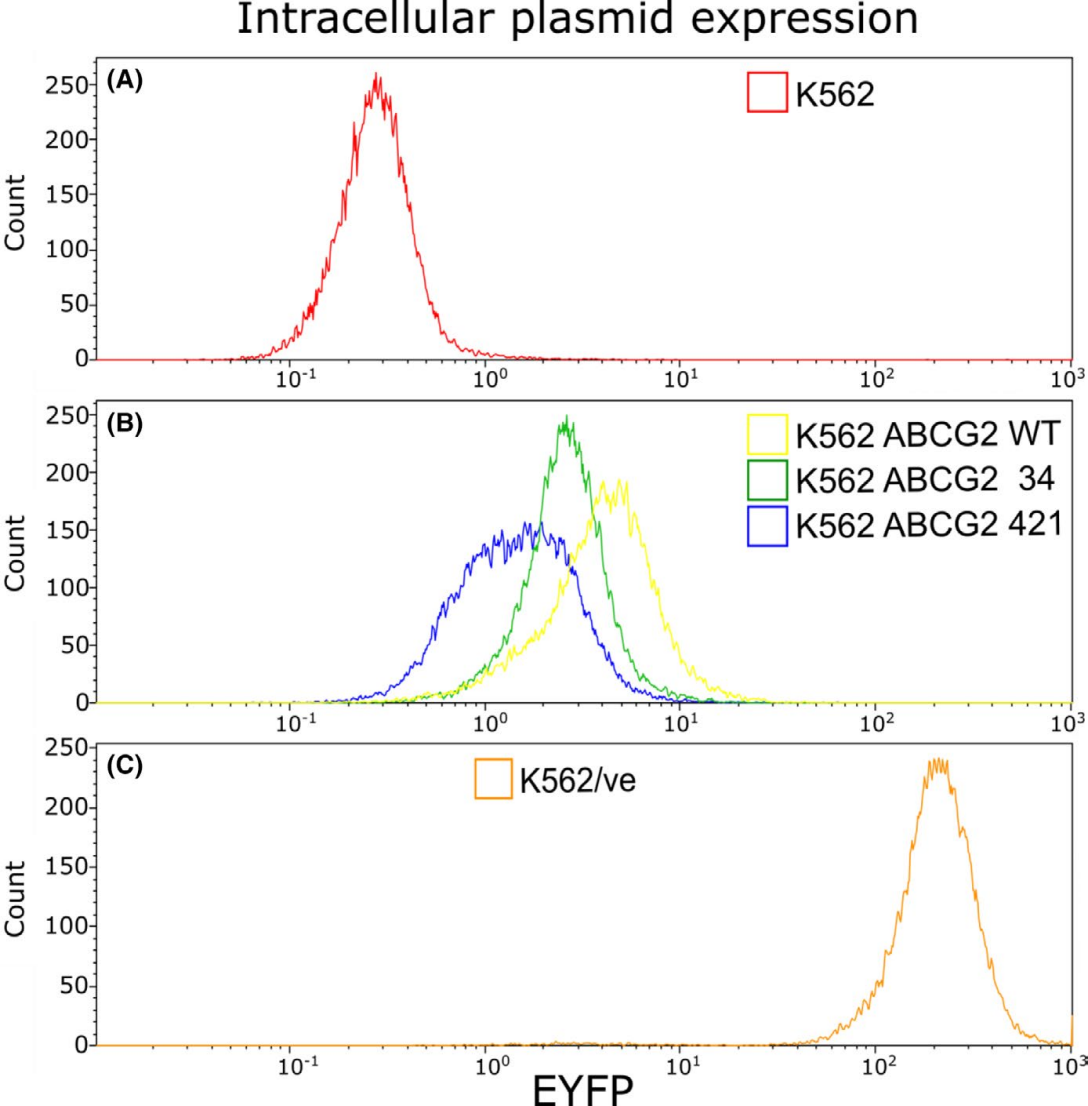
Expression of EYFP in all of the studied cell lines (A‐C). The EYFP expression, which indicates plasmid content inside of the cells, was observed in all ABCG2 transduced cell lines (B). Extremely high EYFP expression was observed in the control cell line K562/ve (C)

In the flow cytometry analysis of the *ABCG2* transduced K562 cell lines confirmed EYFP expression in all transduced cell lines, Figure 1, indicating that plasmids were located inside the cells. A lower EYFP expression was observed in the K562 *ABCG2* 421 (mean fluorescent intensity (MFI) = 1.18) compared with K562 ABCG2 wt (MFI = 4.68) and K562 ABCG2 34 (MFI = 2.87). Despite low EYFP expression, a signal compared to the K562 (MFI = 0.39) was observed, suggesting that the K562 *ABCG2* 421 cell line harbor plasmids even though it is low. The EYFP expression in the control cell line K562/ve was extremely high (MFI = 223), Figure 1B.

**FIGURE 2:**
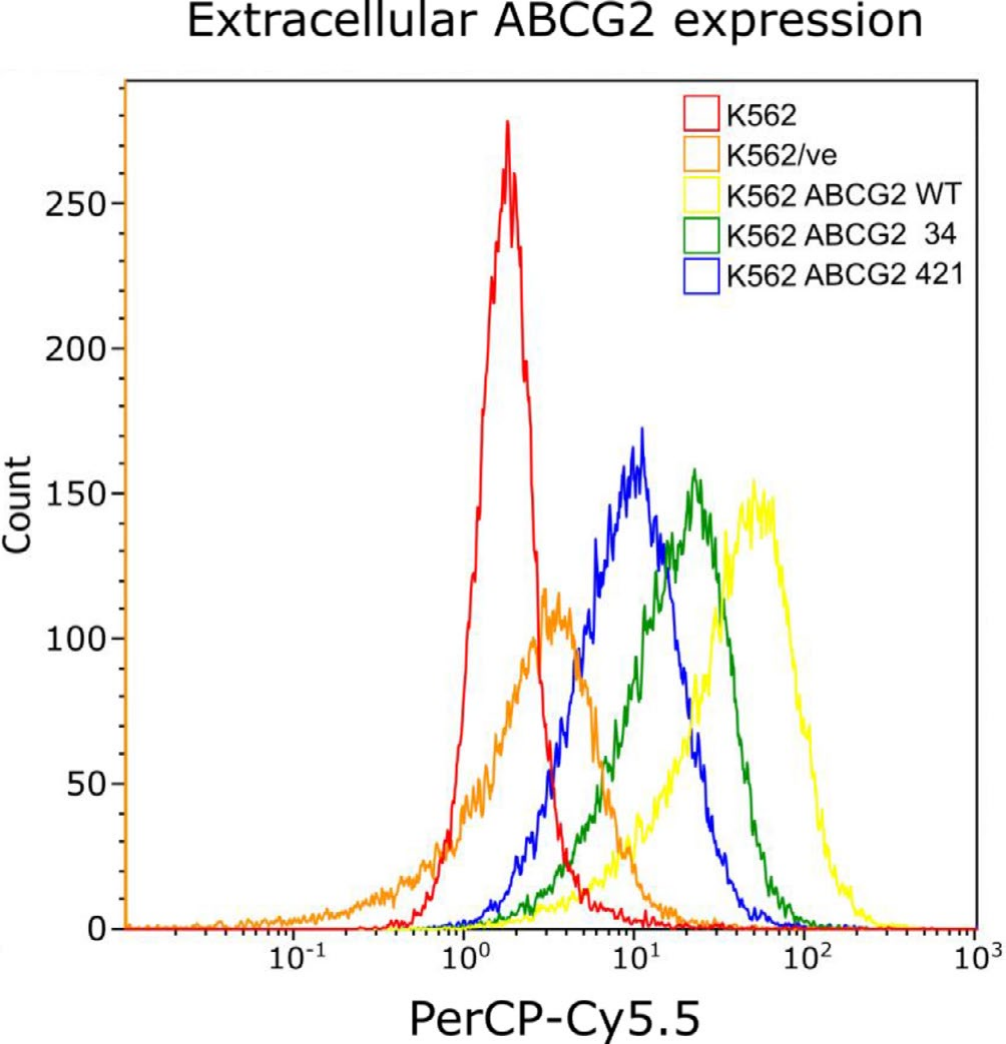
PerCP‐Cy5.5 expression in all of the studied cell lines. The PerCP‐Cy5.5 expression, which corresponds to the extracellular ABCG2 expression, varies between transduced cell lines

The ABCG2 expression, determined based on the PerCP‐Cy5.5 response, was most prevalent in the K562 *ABCG2* wt (MFI = 49.6) and followed in decreasing order in K562 *ABCG2* 34 (MFI = 21.0) and K562 *ABCG2* 421 (MFI = 8.49), Figure 2. The ABCG2 expression normalized to EYFP response was 10.3, 7.0, and 6.5 for K562 *ABCG2* wt, K562 *ABCG2* 34, and K562 *ABCG2* 421, respectively.

### 3.2 Quantification of erlotinib using LC–MS/MS

The performance of the LC–MS/MS method for the quantification of erlotinib was monitored in each run (n = 2). Precision and accuracy for erlotinib in all quality control samples were < 5% and between 92% and 108%, respectively.

### 3.3 Intracellular accumulation of erlotinib

**FIGURE 3:**
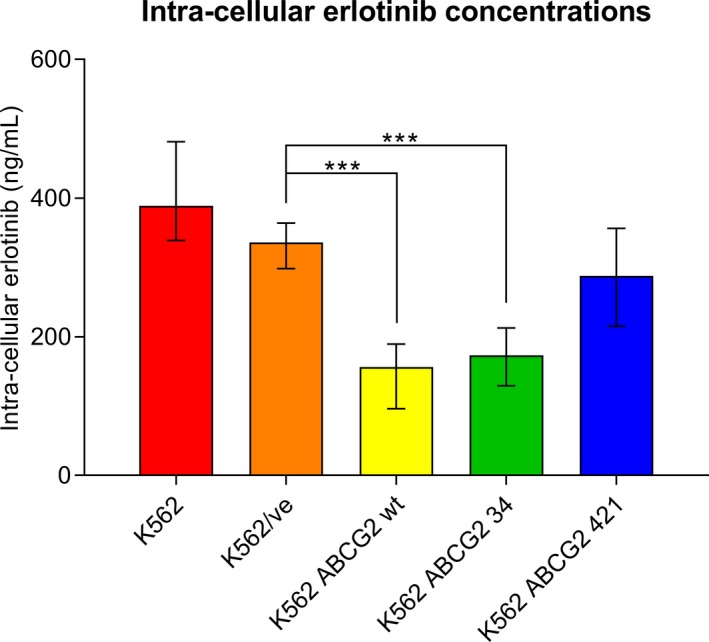
Intracellular erlotinib concentrations measured after incubation with 1 µmol/L erlotinib for 60 minutes in K562 and the K562 recombinant cell lines K562/ve, K562 *ABCG2* wt, K562 *ABCG2* 34, and K562 *ABCG2* 421. The bars illustrate mean erlotinib concentrations with range (n = 6, K562/ve (n = 4)). Statistical difference in intracellular erlotinib concentrations compared to K562/ve was observed in K562 *ABCG2* WT and K562 *ABCG2* 34. Note: ***P < .001

No statistical differences in intracellular erlotinib concentrations were observed between the parental K562 cell line and the K562/ve cell line. All intracellular‐concentrations in ABCG2‐expressing cell lines were compared with K562/ve. Statistical differences were observed between the K562/ve and the K562 *ABCG2* wt (*P* ≤ .001) and K562/ve and K562 *ABCG2* 34 (*P* ≤ .001), Figure 3. The intracellular concentrations for the K562 *ABCG2* 421 were not significantly different from the K562/ve.

**FIGURE 4:**
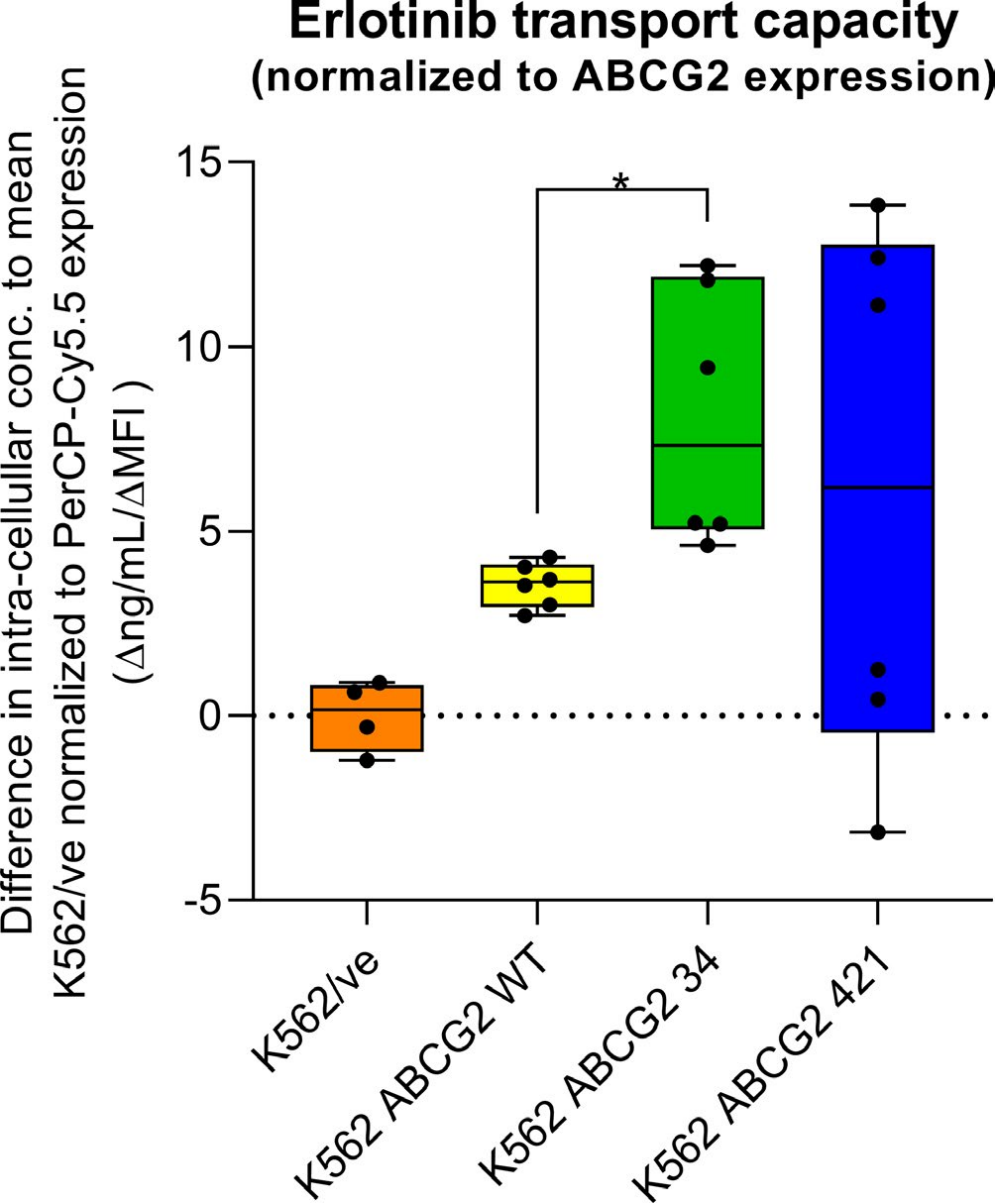
Intracellular erlotinib concentrations normalized to ABCG2 expression were determined from differences in intracellular concentrations between mean K562/ve and each cell line and divided by ABCG2 expression determined from PerCP‐Cy5.5. Differences in transport capacity between K562 *ABCG2* wt and K562 *ABCG2* 34 was analyzed using Student's independent *t* test. Note: *P < .05

To account for variation in ABCG2 expression in the recombinant cell lines, the intracellular erlotinib concentrations were normalized to the ABCG2 expression, Figure 4. The ABCG2 normalized intracellular concentrations were determined from the difference in intracellular concentration of the average K562/ve and a replicate from an ABCG2‐expressing cell line and divided by the PerCP‐Cy5.5 response from the ABCG2‐expressing cell line adjusted for isotype expression (Δ ng/mL/Δ MFI). The K562 *ABCG2* 34 showed a higher erlotinib transport capacity compared to K562 *ABCG2* wt, despite similar intracellular concentrations due to lower ABCG2 expression in K562 *ABCG2* 34 (*P* = .024). The normalized erlotinib transport capacity for K562 *ABCG2* 421 was widely spread and around zero.

## 4 DISCUSSION

In this project, we have studied erlotinib efflux by K562 cells carrying wild‐type ABCG2 and the *ABCG2* SNPs 34 G > A and 421 C > A. The intracellular concentrations in K562 *ABCG2* wt and K562 *ABCG2* 34 were significantly lower compared to the K562/ve indicating that the two cell lines actively transport erlotinib out of the cell, Figure 3. The K562 *ABCG2* 34 exhibited a higher transport capacity compared to K562 *ABCG2* wt, when the intracellular concentrations were normalized to ABCG2 expression, due to lower ABCG2 expression in K562 ABCG2 34, meanwhile similar amounts of erlotinib were transported out of the cells, Figure 4. This finding should be viewed with caution as this might be due to limitations of the modeling system. The intracellular erlotinib concentrations in K562 *ABCG2* 421 did not differ from K562/ve, and when normalized to ABCG2 expression, the ABCG2 transport capacity was widely spread and included negative values, Figures 3 and 4.

The cell line used in this project, the chronic myeloid leukemia K562 cell line, expresses no or very low amounts of ABCG2 and EGFR (http://www.proteinatlas.org).^16^ Therefore, K562 is a suitable model to solely study erlotinib transport as cell survival is not affected.

The control cell line, K562/ve, containing only an empty vector without the ABCG2 gene, exhibited a very high EYFP expression. This was probably due to the more successful transduction of the empty vector as it was smaller, 6.5kb, compared to the other vectors that additionally contained 4.5kb of ABCG2 cDNA. A more even distribution of EYFP expression among the cell lines would probably have been achieved if the size difference between the empty vector and the ABCG2 containing vectors had been smaller.

The two cell lines K562 *ABCG2* wt and K562 *ABCG2* 34 exhibited significantly lower erlotinib intracellular concentrations indicating that erlotinib was actively transported out of the cells. It is in line with previous studies where *ABCG2* 34 showed similar expression levels and transport capacity as wild type in other studied substances.^13^

It has previously been shown that 421C > A results in a lower ABCG2 expression.^12^ Low ABCG2 expression was also observed for K562 *ABCG2* 421 in this study, but it may also be caused by the reduced plasmid content. The results in this study suggest that cells carrying the 421 C > A gene variant are less effective than those with ABCG2 wt and 34G > A to transport erlotinib out of the cell. Our results confirm earlier results that there is no difference between cells carrying the 421C > A and control cell line.^14^ It has also been showed that erlotinib‐treated Japanese patients carrying the ABCG2 421A (MAF = 0.28) have a higher erlotinib steady‐state plasma trough concentration compared to patients carrying the wild type,^21^ possibly caused by reduced erlotinib transport due to altered ABCG2 protein function. A study by Rudin et al,^2^ mainly on a Caucasian population (MAF = 0.08), did not find the same results.^2^ A possible reason for the lack of significance in the study by Rudin et al^2^ could be due to the difference in the allele frequency among the studied populations.

In summary, we have studied the transport of erlotinib in the K562 cell lines transduced with ABCG2 wt and ABCG2 SNPs in this project. We have identified that erlotinib efflux is affected by SNPs in ABCG2 which leads to variation in intracellular concentrations. The results obtained in this study indicate that K562 *ABCG2* wt and K562 *ABCG2* 34 actively transports erlotinib by efflux while K562 *ABCG2* 421 exhibits a reduced erlotinib transport capacity. ABCG2 polymorphism can, therefore, be considered as a possible contributor to the interindividual variation observed in erlotinib pharmacokinetics and toxicity.

## DISCLOSURE

The authors declare no conflicts of interest.

## AUTHOR CONTRIBUTIONS

Participated in research design: Green, Vikingsson, Svedberg, and Jacobs. Conducted experiments: Svedberg and Jacobs. Performed data analysis: Svedberg and Jacobs. Wrote or contributed to the writing of the manuscript: Green, Vikingsson, Svedberg, and Jacobs.
